# In this month’s *Bulletin*

**DOI:** 10.2471/BLT.15.000415

**Published:** 2015-04-01

In the editorial section, Jamie Bartram et al. (210) highlight a recent survey of 54 countries showing that thousands of health facilities do not have water, soap and/or toilets. Haik Nikogosian and Vera Luiza da Costa e Silva (211) mark the tenth anniversary of WHO's first global health treaty – the Framework Convention on Tobacco Control.

Keiji Fukuda (212) describes the focus of this year’s World Health Day: food safety. In an interview, Antoine Andremont (217–218) focuses on the intersection of measures to improve food safety and decrease antimicrobial resistance.

Fiona Fleck (215–216) reports on efforts to get accurate tests for the Ebola virus closer to the point-of-care.

## Lao People’s Democratic Republic

## Understanding antibiotic use

Fabrice Quet et al. (219–227) survey prescribing behaviours of doctors working in public hospitals.

## Mexico

## Is task-shifting an option?

Claudia Diaz Olavarrieta et al. (249–258) compare nurses’ and doctors’ provision of medical abortion services.

## United Republic of Tanzania

## Mid-course corrections

Leonard EG Mboera et al. (271–278) describe lessons learnt from monitoring the national health sector plan.

## Global

## Market interventions for tuberculosis medicines

Nimalan Arinaminpathy et al. (237–248) study the Global Drug Facility’s impact on tuberculosis medicine prices over the last decade. Kaspars Lunte et al. (279–282) track recent price reductions for second-line treatments.

## Surviving the first six weeks

Étienne V Langois et al. (259–270) review the evidence for equitable access to postnatal care services.

## Difficult decisions on essential medicines

Nicola Magrini et al. (283–284) outline challenges facing the next essential medicines committee.

## Making sense of global estimates

Scott A McDonald et al. (228–236) propose methods for getting from global to national burden of disease estimates

